# KLF1 Exerts Pro‐Tumour Role in Liver Cancer via Inhibiting ACSL4/LPCAT3‐Regulated Ferroptosis

**DOI:** 10.1111/jcmm.71033

**Published:** 2026-02-08

**Authors:** Zhihui Chen, Changyan Zhang, Jialin Yang, Yong Peng

**Affiliations:** ^1^ Department of Hepatobiliary Surgery Beijing Anzhen Nanchong Hospital of Capital Medical University & Nanchong Central Hospital Nanchong Sichuan China

**Keywords:** ACSL4, ferroptosis, KLF1, primary hepatocellular carcinoma, targeted therapy

## Abstract

Kruppel‐like factors (KLFs) constitute a crucial family of transcription factors that are engaged in a variety of biological processes, such as erythropoiesis as well as liver development. A growing body of research underscores the increasing importance of the KLF family in the context of hepatocellular carcinoma (HCC). Despite this, the exact function of KLF1 within HCC remains unclear. Our study demonstrates a significant upregulation of KLF1 expression in tumour samples from HCC patients compared to normal liver tissue, with higher expression levels strongly correlating with poorer survival outcomes. Notably, in vitro experiments have shown that KLF1 enhances liver cancer cell proliferation by inhibiting ferroptosis, and this inhibition is negatively correlated with the transcription levels of fatty acid synthase 4 (ACSL4). These findings suggest that KLF1 may exert its oncogenic potential by repressing ferroptosis through the inhibition of ACSL4 transcription. Further mechanistic investigations reveal that KLF1 inhibits ACSL4 expression via transcriptional repression and suppresses ferroptosis through the ACSL4/LPCAT3 axis, ultimately promoting HCC tumour growth as well as its advancement. In conclusion, KLF1 is essential for onset as well as development in HCC through inhibiting ACSL4 transcription along with ferroptosis, thereby presenting novel therapeutic targets for HCC treatment.

## 1 Introduction

Hepatocellular carcinoma (HCC), a malignant tumour with a high incidence and mortality rate worldwide, often develops from chronic liver diseases such as hepatitis virus infections and cirrhosis. The progression of HCC is intricate, involving disruptions in various molecular pathways and cellular signalling, leading to the uncontrolled growth in liver tissue as well as tumour formation [1]. During its early stages, liver cancer typically does not exhibit symptoms, and the absence of effective early screening methods results in most patients being diagnosed at advanced stages, which significantly hampers treatment effectiveness [2, 3]. Although there have been notable advancements in surgical interventions, radiotherapy, chemotherapy and targeted therapies, liver cancer continues to present significant challenges in clinical management because of the high recurrence rate as well as drug resistance [4, 5, 6]. A deeper understanding of the mechanisms driving liver cancer will aid in identifying novel therapeutic targets, advancing personalised and targeted treatments and ultimately enhancing patient outcomes as well as overall well‐being.

Kruppel‐like factors (KLFs), a family of transcription factors, are defined by their zinc finger domains, are crucial regulators involved in a wide range of biological activities, such as proliferation, differentiation, apoptosis as well as metabolism [7, 8]. KLF family comprises 17 members, which are grouped into three categories: KLF1 to KLF4, KLF5 to KLF8 and KLF9 to KLF17 [9]. These factors regulate gene transcription primarily through their interaction with specific DNA motifs. Research has shown how KLFs are involved in regulating liver cancer progression, with each member exerting distinct effects. For instance, KLF2 is recognised for functioning as a cancer inhibitor during liver cancer [10], whereas KLF13 enhances development linked to HCC by modulating cholesterol synthesis through the transcription of HMGCS1 [11]. Among the KLFs, KLF2, KLF13 and others have been shown to regulate the progression of liver cancer. However, as a member of the KLF family, KLF1 is primarily involved in regulating erythropoiesis and haemoglobin synthesis, and its role in liver cancer has not been studied. Consequently, investigating KLF1's involvement in hepatocellular carcinoma may provide crucial understanding regarding cellular processes driving liver cancer and assist in developing novel therapeutic approaches.

Ferroptosis is a novel form of programmed cell death, primarily triggered by the accumulation of iron‐dependent lipid peroxides. It has unique biological characteristics, and previous studies have shown that ferroptosis plays an important role in the development and progression of liver cancer [12]. Unlike conventional forms of cell death, ferroptosis is initiated through an excess in intracellular iron ions, which stimulate lipid peroxidation, resulting in membrane damage followed by cell death. Regulation in ferroptosis involves multiple molecular pathways, particularly those associated with lipid metabolism [13]. Lipid peroxidation represents a key factor in ferroptosis, primarily by regulating fatty acid synthesis, transport and oxidation. Key proteins, including acyl‐CoA synthetase long‐chain family member 4 (ACSL4) as well as LPCAT3, serve a vital function within this process. ACSL4 activates fatty acids, promoting lipid peroxidation in cell membranes, while LPCAT3 regulates phospholipid synthesis and modification, amplifying lipid peroxidation and advancing ferroptosis [14]. Emerging studies suggest that KLF1, as a transcription factor, may influence ferroptosis, presenting potential molecular targets for treating related diseases [15].

In this research, we explored the oncogenic function of KLF1 in both hepatocellular carcinoma (HCC) cell lines and the xenograft mouse model we established.

## 2 Materials and Methods

### 2.1 An Investigation Into KLF1 Levels, Survival Outcomes, Along With Clinical Characteristics

We employed Kaplan–Meier curve analysis to examine the association between KLF1 mRNA levels with key outcomes, such as overall survival (OS), progression‐free survival (PFI), disease‐free survival (DFI), as well as disease‐specific survival (DSS). This assessment utilised R packages “survival,” “survminer,” “limma,” and “ggpubr.”

### 2.2 Antibodies and Reagents

In this study, the following antibodies were utilised: anti‐KLF1 (A10581, abclonal), anti‐xCT (A2413, abclonal), anti‐GPX4 (A11243, abclonal), anti‐β‐actin (RP02968LQ, abclonal), anti‐P53 (A22449, abclonal) and Ki67 (A20018, abclonal). Additionally, ACSL4 (A20414, abclonal) and LPCAT3 (A17604, abclonal) were also employed.

Human HCC cell lines, including Huh7, SNU‐447, THLE‐2, HLF and MHCC97‐H, had been procured via a Cell Bank of the Shanghai Institute of Biochemistry plus Cell Biology (Shanghai, China). These lines grew on DMEM (Genom, Hangzhou, China) supplemented by 10% fetal bovine serum (Thermo Fisher Scientific) as well as 1% penicillin/streptomycin (Biosharp, Anhui, China), then cultured in a 37°C incubator containing 5% CO_2_.

To induce cell death, inhibitors such as Z‐VAD (10 μm), DSF (0.5 μm), IM‐54 (3 μm), Nec‐1 (10 μm), Erastin (5 μm) and Fer‐1 (1 μm) were applied to the HCC cells as necessary [16].

### 2.3 Cell Culture and Transfection

Human liver cancer cell lines (all obtained from the American Type Culture Collection, ATCC, Manassas, Virginia, USA) were cultured in medium containing 10% fetal bovine serum (FBS, Yeasen Biotechnology Co. Ltd., Shanghai, China) and 1% penicillin–streptomycin (Sigma‐Aldrich, Merck KGaA, Darmstadt, Germany) in a humidified incubator with 5% CO_2_ at 37°C. Liver cancer cells (2 × 10^5^ cells per well) were seeded into 6‐well plates and cultured overnight until cell confluence reached 50%–70%. shRNA (GenePharma Co. Ltd., Shanghai, China) and overexpression (OE) plasmids (Hanbio Biotechnology, Shanghai, China) were transfected into cells using Lipofectamine 3000 (Invitrogen, Carlsbad, California, USA) in serum‐free medium. After 6 h of transfection, the cells were cultured for 30 h in complete medium.

### 2.4 Cell Viability Test

Cell viability was assessed using the Cell Counting Kit‐8 (CCK‐8) (Topscience, Shanghai, China). Cells, plated with 5000 cells in each compartment within 96‐well containers, received various amounts of fatostatin along with additional compounds during a 24‐h interval. Following the manufacturer's instructions, 10 μL of CCK‐8 solution had been dispensed into every compartment, and cells underwent incubation for 1 h at 37°C. After incubation, absorbance readings were taken at 450 nm with a Multimode Plate Reader (PerkinElmer, Germany).

### 2.5 Colony‐Formation Assay

To perform the colony‐formation assay, 500 cells were placed in each compartment of six‐well dishes, followed by incubation for about 2 weeks. Colonies underwent fixation using 4% formaldehyde solution (Biosharp, Anhui, China), followed by treatment with 0.1% crystal violet (G1014, Servicebio, Wuhan). Afterward, selected colonies underwent photography followed by measurement.

### 2.6 EdU‐DNA Synthesis Assay

Cell growth underwent evaluation with EdU Cell Proliferation Kit with Alexa Fluor 555 (MA0425, Meilun, Dalian) after treating the cells with varying concentrations of fatostatin for 24 h. The pre‐warmed EdU working solution was applied to the treated cells for 2 h for EdU labeling. After removing the medium, the cells were fixed for 30 min. Following fixation, 500 μL of Click reaction solution was added into every compartment, followed by incubation in the dark under ambient temperature for 3 min. During the final step, 1 mL Hoechst 33342 dye was introduced, followed by a 10‐min incubation with the specimens in ambient conditions, away from light. Once the dyeing process was completed, photographs were captured with the confocal imaging device (Olympus BX51, Japan).

### 2.7 RNA‐Seq

Total RNA isolation occurred with the TRIzol reagent (Invitrogen) following exposure of the cultures to varying quantities of fatostatin over a 24‐h duration. Sequencing was performed on the NovaSeq 6000 system, utilising the NovaSeq S4 reagent kit. Differentially expressed gene (DEG) analysis between the two groups involved conducting the DESeq R package (1.18.1), with criteria specified by |log_2_(FoldChange)| > 1, along with a corrected *p* value below zero point zero five. Enrichment analyses for Gene Ontology (GO), KEGG, GSEA, as well as Reactome were conducted using the R package “clusterProfiler.” Genes having corrected *p* values as well as raw *p* values below zero point zero five underwent identification as prominently upregulated.

### 2.8 Wound‐Healing Assay

Wounds underwent creation through abrasion to a monolayer cultured within 6‐well dishes using a 200 μL micropipette nozzle. To eliminate the influence of cell proliferation, they underwent cultivation within a culture solution supplemented with only 1% FBS. Following treatment using varying doses in fatostatin, wound size underwent capture during both 0 h and 48 h. Dotted lines were used to mark the boundaries of the wound. The wound width was measured with ImageJ (version 1.53e), and percentage wound recovery was then determined as well as assessed.

### 2.9 Transwell Assay

To conduct the Transwell assay, Transwell chambers with polycarbonate membranes coated with Matrigel (R&D, USA) were used. Cells, treated with different concentrations of fatostatin or other reagents, were seeded into the upper chamber with 200 μL of serum‐free medium, while 600 μL of medium containing 10% FBS underwent placement into the bottom chamber to serve as an attractant. Following a 24‐h culture at 37°C with 5% CO_2_ and subsequent treatment using 4% formaldehyde, adherent structures at the bottom surface in Transwell chamber underwent staining using zero point 5% violet dye. Images were obtained and cell counts were taken using a fluorescence instrument (Olympus BX51, Japan).

### 2.10 Western Blotting Analysis

Proteins underwent separation using SDS‐PAGE, followed by subsequently being transferred to PVDF membranes (Millipore, Germany). After blocking the blot, it underwent incubation in primary antibody solution, followed by secondary immunoglobulin (Proteintech, Wuhan, China). Protein images underwent acquisition with the ChemiDoc Touch apparatus (Bio‐Rad, USA). Relative protein quantities underwent normalisation against β‐actin.

### 2.11 Lipid Peroxidation Level Detection

The levels of malondialdehyde (MDA) within HCC samples underwent quantification using the Lipid Peroxidation MDA Assay Kit (S0131S, Beyotime, China). To determine the relative MDA concentration, total protein amount underwent measurement with a BCA Kit (P0012, Beyotime). Lipid reactive oxygen species (ROS) levels were assessed within the samples through the C11 BODIPY 581/591 fluorescent probe (Mao‐kangbio, MX5211–1 MG). HCC samples underwent incubation in 5 μM C11‐BODIPY during 30 min in 37°C, followed by image acquisition with a confocal imaging system. Reduced dye underwent quantification at Ex/Em = 581/591 nm (Texas Red filter), while oxidised dye underwent measurement at Ex/Em = 488/510 nm (FITC filter).

### 2.12 Measurement of Fe^2+^ Content

Cells were plated at a density of 1 × 10^5^ cells/mL in a 12‐well culture plate. Intracellular Fe^2+^ levels were determined using the Ferro‐orange kit (#ab83366, Abcam, Cambridge, MA, USA) according to the manufacturer's guidelines. After treatment, the cells were incubated in serum‐free medium for 4 h and subsequently stained using Ferro‐orange during 30 min in 37°C. Fluorescence intensity underwent measurement with the microplate spectrophotometer (#1681150, Bio‐Rad, Hercules, CA, USA), with the appropriate excitation and emission wavelengths to quantify the fluorescence intensity.

### 2.13 Glutathione Assays

The GSH levels within HCC cells underwent determination with the Glutathione Assay Kit (S0053, Beyotime). To derive relative GSH concentration, protein levels underwent quantification with a BCA Kit (P0012, Beyotime).

### 2.14 Chromatin Immunoprecipitation Combined With Quantitative PCR (ChIP‐qPCR) Assay

Chromatin underwent isolation in HCC samples and fragmented enzymatically. Immunoprecipitation was carried out using an anti‐KLF1 antibody along with the Pierce Agarose ChIP Kit, following the manufacturer's guidelines. As a negative control, rabbit IgG was included. Immunoprecipitated chromatin underwent real‐time qPCR analysis using SYBR Premix DimerEraser (Perfect Real Time) (RR091A, Takara). Each sample was tested across three replicates, with the results obtained for both KLF1 IP alongside mock IP represented by enrichment in comparison with total DNA. To confirm the consistency of the results, the ChIP‐qPCR procedure was performed twice.

### 2.15 Transmission Electron Microscopy (TEM)

Biological tissues underwent harvesting followed by fixation using a reagent with two point 5% paraformaldehyde (Servicebio). The samples underwent postfixation with 1% osmium tetroxide, followed by dehydration. Following specimen inclusion within Epon, the sections were stained with uranyl acetate. Imaging was performed using a transmission electron microscope (Hitachi HT7700, Tokyo, Japan).

### 2.16 Immunohistochemistry (IHC)

The 5 μm thick specimen slides underwent dewaxing, rehydration, followed by antigen retrieval in EDTA buffer. Next, the samples were incubated in 3% H₂O₂ for 10 min, followed by blocking using 5% BSA for 45 min. The sections were then exposed to a mixture of rabbit primary antibodies (anti‐KLF1, anti‐P53, anti‐CyclinD, anti‐Ki67, anti‐ACSL4, anti‐LPCAT3) and incubated overnight at 4°C. On the subsequent day, slides were treated with goat anti‐rabbit IgG secondary antibody for 30 min. Afterward, they underwent staining using 3,3′‐diaminobenzidine (DAB) and counterstaining with eosin. Specimens underwent fixation, dehydration and drying before being examined under a microscope. For analysis, 10 arbitrarily chosen areas on each slide were evaluated and semi‐quantitatively analysed with ImageJ software (version 1.8.0).

### 2.17 Quantitative Real‐Time PCR

Following isolating RNA obtained out of HCC cultures, cDNA was generated using the PrimeScript RT Reagent Kit (RR047A, TaKaRa, Japan). To measure mRNA levels, SYBR Premix Ex Taq II (RR820A, TaKaRa, Kusatsu, Japan) along with the Bio‐Rad CFX Manager 2.1 real‐time PCR system (Bio‐Rad, Hercules, CA, USA) underwent utilisation. mRNA levels underwent analysis using a comparative Ct method, with β‐actin functioning in place as a housekeeping reference marker.

### 2.18 Nude Mice Xenograft

Male BALB/c nude mice, 4 weeks old together with SPF grade, had been sourced through Hunan Silaijingda Experimental Animal Co. Ltd. (Changsha, China), with the quality certificate number 430727221101364576. These mice resided within an environment controlled for conditions with a 12‐h light/dark schedule, receiving unlimited provision for both food and water. Following a 7‐day acclimatisation period, each mouse received a subcutaneous injection of 1 × 10^7^ HCC cells in the right axillary region. Tumour length and width were measured 14 days post‐treatment, and tumour volume was determined using the formula: Volume = length × width^2^/2.

### 2.19 Statistical Analysis

Statistical analyses occurred with GraphPad Prism 9.0 (San Diego, CA, USA). The results appeared represented by the mean ± SEM. A *p*‐value below zero point zero five qualifies as meaningful statistically.

## 3 Results

### 3.1 KLF1 Exhibits High Levels Within HCC, Correlating to Poor Outcomes

**FIGURE 1:**
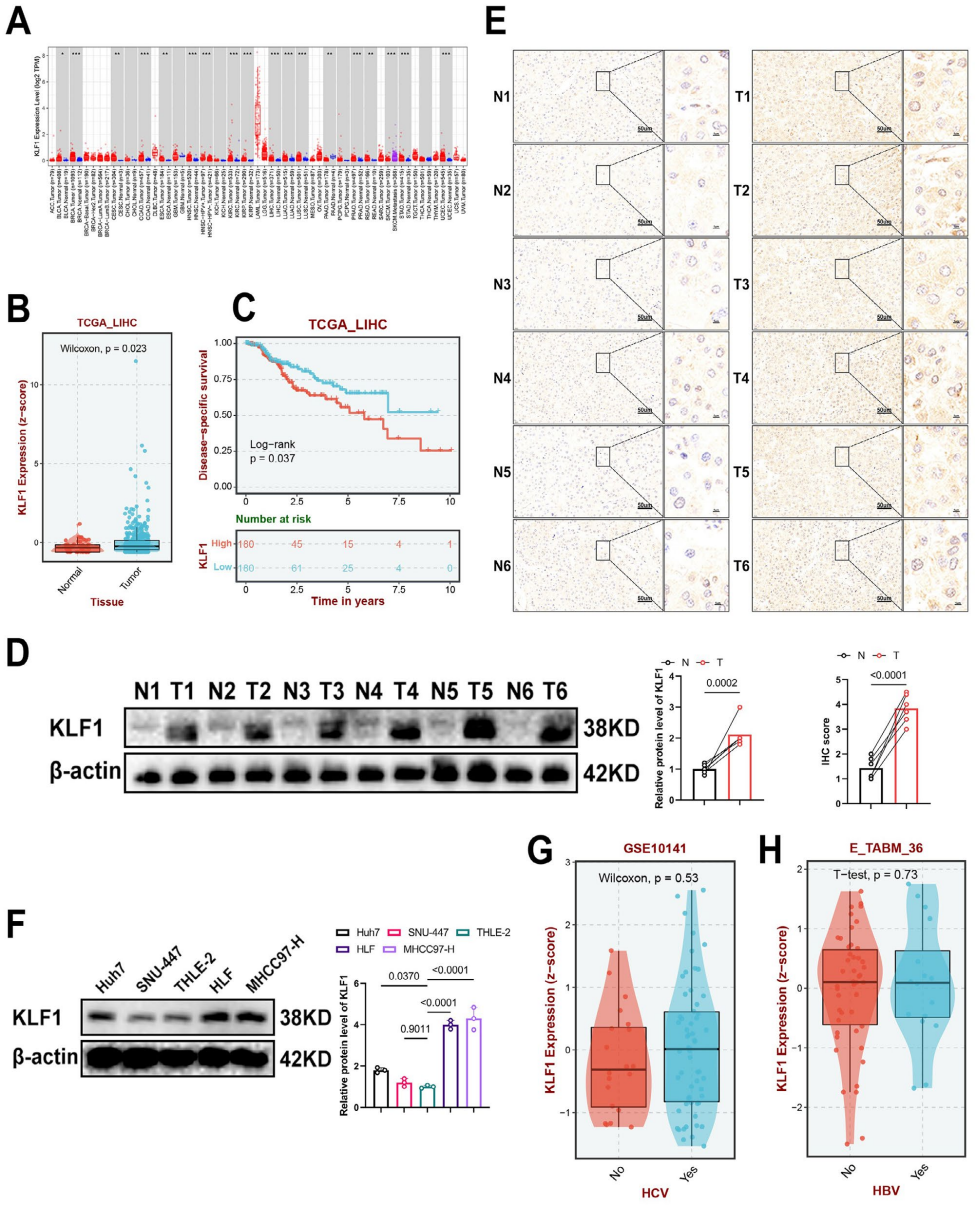
KLF1 is highly expressed in liver cancer and correlates with poor prognosis. (A, B) Public database analysis reveals that KLF1 appears notably overexpressed within liver cancer. (C) Kaplan–Meier survival analysis shows correlation of KLF1 levels with disease‐specific survival. The red line indicates high expression of KLF1, whereas the blue line corresponds to low expression. (D, F) Western blot assays underwent analysis for measuring KLF1 protein expression within liver cancer tissues (D) and cell lines (F). (E) Representative images and quantitative analysis of KLF1 expression within liver cancer samples, as well as neighbouring non‐cancerous samples, performed through immunohistochemistry (IHC). (G, H) Correlation examination linking KLF1 levels within liver cancer with HBV (G) and HCV (H). Data underwent presentation as mean ± SEM. *p*‐values underwent determination using two‐tailed independent *t*‐tests. Significance is assessed for *p*‐values below 0.05, 0.01, 0.001, as well as 0.0001, corresponding by *, **, ***, and **** respectively.

To assess KLF1 expression across cancer types, an analysis took place utilising TCGA data. Findings indicated KLF1 levels showed upregulation within liver cancer, adrenal cortical carcinoma and low‐grade gliomas, while it was downregulated in breast cancer, cervical cancer, along with various additional tissue types (Figure 1A,B). Furthermore, Kaplan–Meier survival analysis showed an association linking increased KLF1 levels to significantly reduced disease‐specific survival (*p* = 0.037) (Figure 1C), suggesting that KLF1 may act as a negative prognostic marker associated with an increased risk of disease progression. We also performed WB and IHC assays on clinical patient samples, which revealed elevated KLF1 levels within HCC samples in comparison with nearby non‐cancerous specimens (Figure 1D,E). Finally, WB analysis was conducted on several cell lines, including Huh7, SNU‐447, THLE‐2, HLF and MHCC97‐H. The data revealed that KLF1 expression was most prominently increased in HLF and MHCC97‐H liver cancer cells compared to THLE‐2 cells, with smaller increases also observed in Huh7 and SNU‐447 liver cancer cells (Figure 1F). Given the results, HLF together with MHCC97‐H had been chosen to undergo further analysis. It is known that Huh7 and SNU‐447 cells are susceptible to HBV or HCV infection, which may drive malignant progression. Further analysis of TCGA data revealed no notable variation of KLF1 expression in specimens harbouring or lacking HBV and HCV infection (Figure 1G,H). This suggests that KLF1 may regulate liver cancer progression driven by non‐chronic hepatitis.

### 3.2 Overexpression of KLF1 Enhances In Vitro Proliferation as Well as Migration in HCC Cultures

**FIGURE 2:**
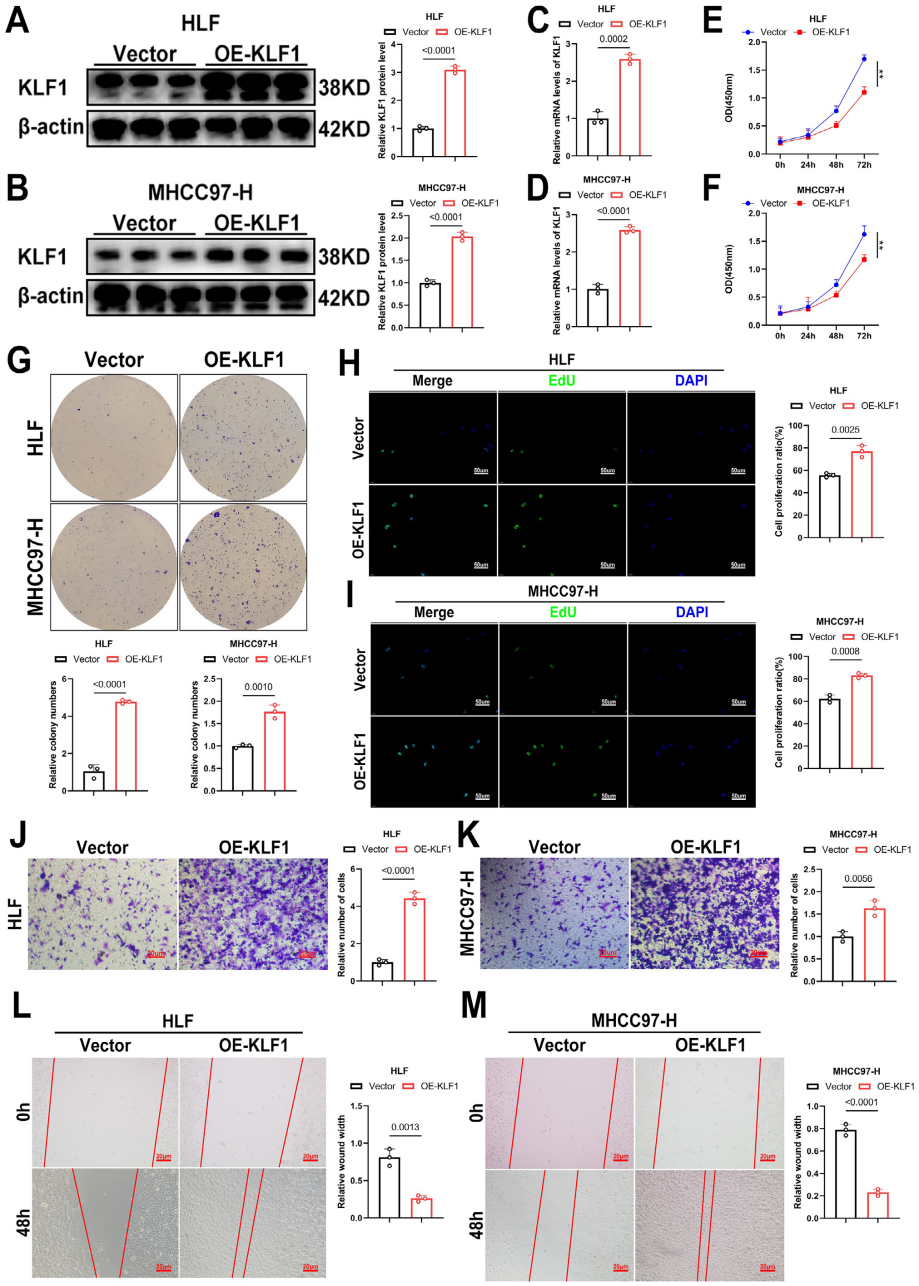
KLF1 overexpression enhances in vitro growth as well as movement in HCC tumours. (A, B) Lentiviral‐mediated delivery confirmed KLF1 upregulation in HCC tumorigenic cells, followed by Western blot analysis to quantify the relative expression levels (*n* = 3). (C, D) KLF1 expression levels were assessed by RT‐qPCR after lentiviral‐mediated KLF1 overexpression (*n* = 3). (E–I) Cell proliferation was evaluated through CCK‐8, colony formation, and EdU assays following KLF1 upregulation. (J–M) The migratory capacity of KLF1‐overexpressing cells was analysed via Transwell as well as wound healing assays. Data are presented as mean ± SEM, and *p*‐values underwent calculation using two‐tailed independent *t*‐tests.

To further investigate KLF1's function within HCC, we successfully generated HCC cells with KLF1 overexpression and verified the efficiency of this overexpression through WB and RT‐qPCR (Figure 2A–D). Subsequently, KLF1's impact in HCC progression underwent examination. Findings from CCK‐8, EdU, as well as colony formation assays revealed KLF1 overexpression greatly enhancing tumour growth potential (Figure 2E–I). Furthermore, Transwell as well as wound healing assays revealed KLF1 overexpression notably enhancing motility in HCC cells (Figure 2J–M). Collectively, the data suggest that KLF1 overexpression enhances both growth as well as movement within HCC malignancies.

### 3.3 KLF1 Knockdown Reduces In Vitro Growth as Well as Movement in HCC Cells

**FIGURE 3:**
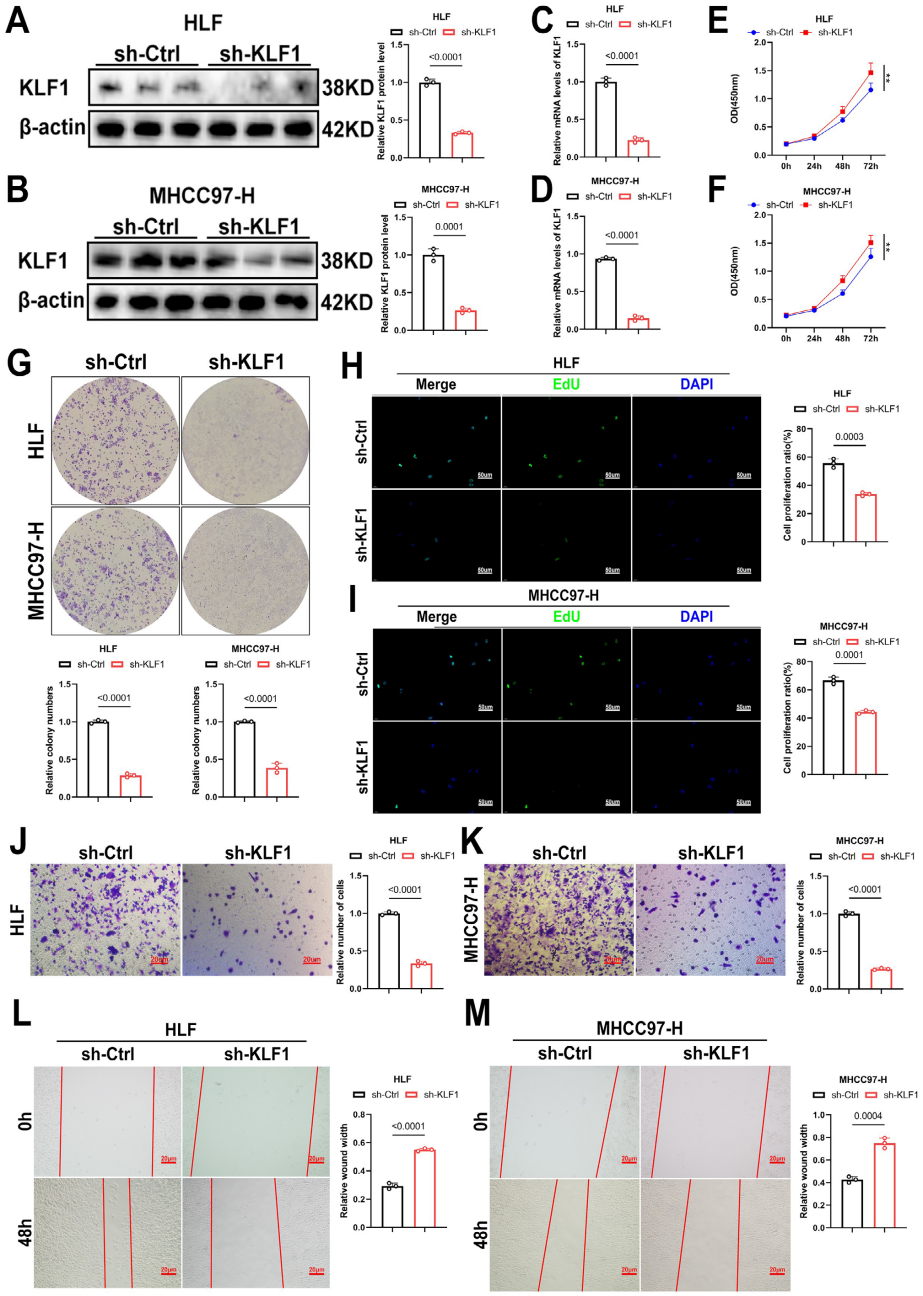
Suppression in HCC tumour growth as well as migration was achieved through KLF1 knockdown in vitro. (A, B) After performing lentivirus‐mediated knockdown, Western blot analysis confirmed the expression of KLF1 (*n* = 3). (C, D) Following KLF1 knockdown with lentivirus, the relative expression of NR4A1 was assessed through RT‐qPCR (*n* = 3). (E–I) Cell proliferation after NR4A1 knockdown underwent assessment through CCK‐8, colony formation, as well as EdU assays (*n* = 3). (J–M) Cell migration following NR4A1 knockdown was examined with Transwell and wound healing assays (*n* = 3). Data are reported as mean ± SEM, while *p*‐values underwent calculation using two‐tailed independent *t*‐tests.

To investigate KLF1's role, we generated HCC cells with KLF1 knockdown and verified the knockdown efficiency through WB and RT‐qPCR (Figure 3A–D). Subsequently, we examined its effect on the progression of HCC. The results of CCK‐8, EdU, as well as colony formation assays demonstrated how KLF1 silencing notably impaired HCC cell growth potential (Figure 3E–I). In addition, Transwell and wound healing assays showed marked decreases in the migratory potential of HCC cells after KLF1 knockdown (Figure 3J–M). Overall, the findings indicate KLF1 knockdown inhibits proliferation as well as migration in HCC.

### 3.4 KLF1 Inhibits Ferroptosis in HCC Cells

**FIGURE 4:**
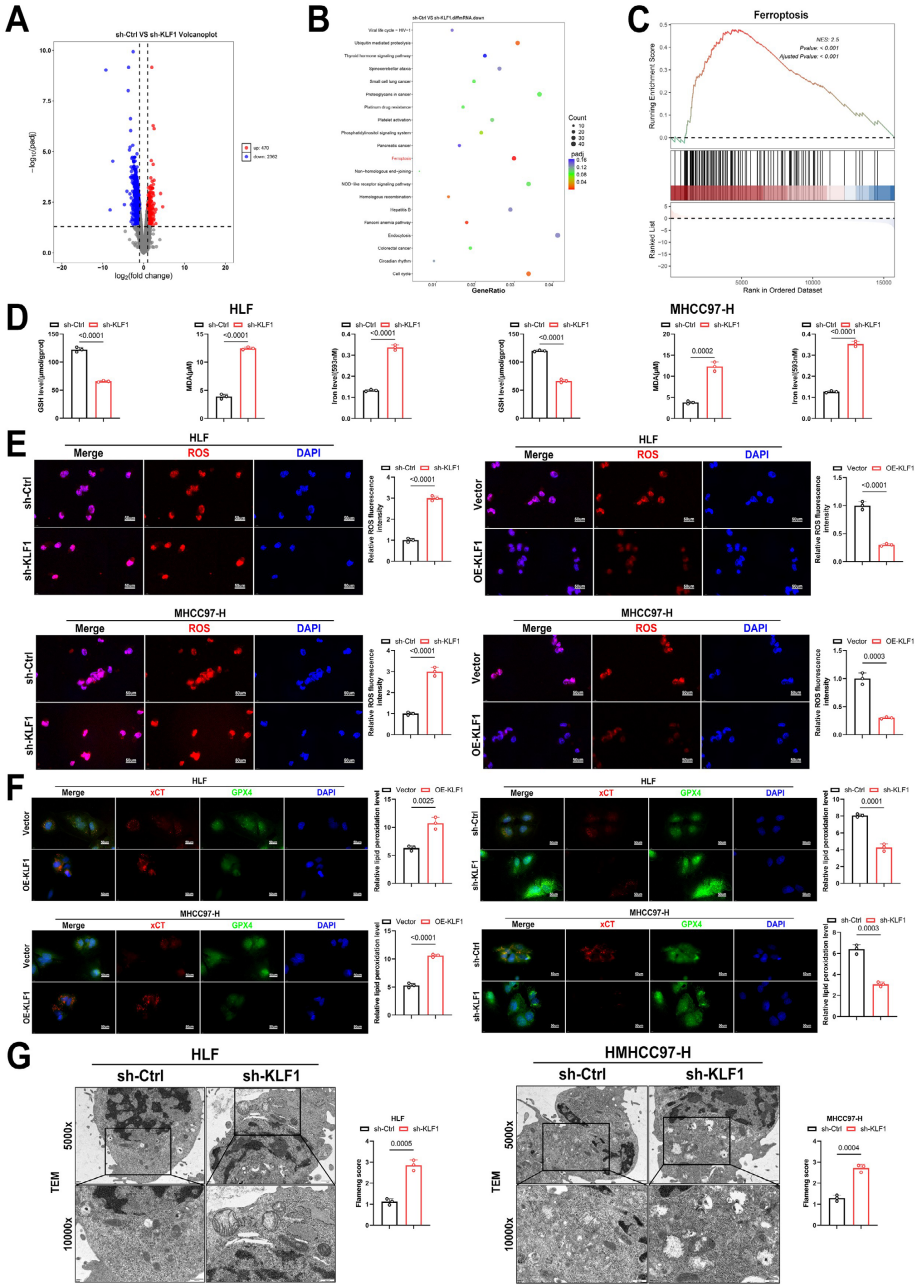
KLF1 inhibits ferroptosis in HCC cells. (A) Volcano plot indicates 470 transcripts were elevated, while 2362 transcripts were suppressed following KLF1 knockdown. (B) KEGG signalling pathway enrichment analysis was conducted using RNA sequencing on the sh‐KLF1 and sh‐Ctrl groups in HLF cells. (C) Gene Set Enrichment Analysis revealed significant association with Kyoto Encyclopedia of Genes and Genomes (KEGG) “Ferroptosis” pathway. (D) GSH, MDA, as well as iron were measured within HCC cells (*n* = 3). (E) Representative ROS detection and its quantitative analysis were performed in HCC cells (*n* = 3). (F) Representative C11 BODIPY staining images of HCC cells are shown (*n* = 3). (G) Representative images obtained through transmission electron microscopy in HCC cells are shown (*n* = 3). Data are shown as mean ± SEM. *p*‐values underwent calculation through two‐tailed independent *t*‐tests.

In order to explore the mechanism underlying the effects of KLF1, transcriptome sequencing was performed on sh‐KLF1 and sh‐Ctrl HLF cells. The volcano plot analysis identified 470 genes with increased expression and 2362 genes with decreased expression (Figure 4A). Both KEGG enrichment and GESA analyses suggested that KLF1's activity is linked to ferroptosis (Figure 4B,C). Ferrous ions, GSH and MDA are widely recognised as key markers of ferroptosis. Upon examining HCC cells, we found that KLF1 knockdown led to elevated levels of ferrous ions as well as MDA, whereas GSH concentrations decreased, indicating that KLF1 knockdown promotes ferroptosis in HCC cells (Figure 4D). ROS (reactive oxygen species) are highly reactive molecules that play a crucial role in ferroptosis by promoting lipid peroxidation, which leads to cellular damage and ultimately triggers ferroptotic cell death. We also assessed intracellular ROS levels, showing that KLF1 overexpression decreased ROS levels, while its knockdown produced the opposite effect (Figure 4E). Lipid peroxidation is the process by which ROS cause oxidative damage to cell membrane lipids, and the C11 BODIPY assay can be used to detect lipid peroxidation levels, serving as an indicator of ferroptosis. C11 BODIPY staining revealed that KLF1 overexpression lowered lipid peroxidation levels, whereas knockdown led to an increase in lipid peroxidation (Figure 4F). In the process of ferroptosis, mitochondrial shrinkage, reduced or disappeared cristae, and increased membrane density are key structural changes that are closely associated with the progression of ferroptosis. TEM analysis demonstrated that knockdown of KLF1 intensified mitochondrial damage in HCC cells (Figure 4G). In conclusion, the findings suggest KLF1 exerts protective effects against ferroptosis within HCC cells.

### 3.5 Regulation of Ferroptosis in HCC Cells Can Restore KLF1 Function

**FIGURE 5:**
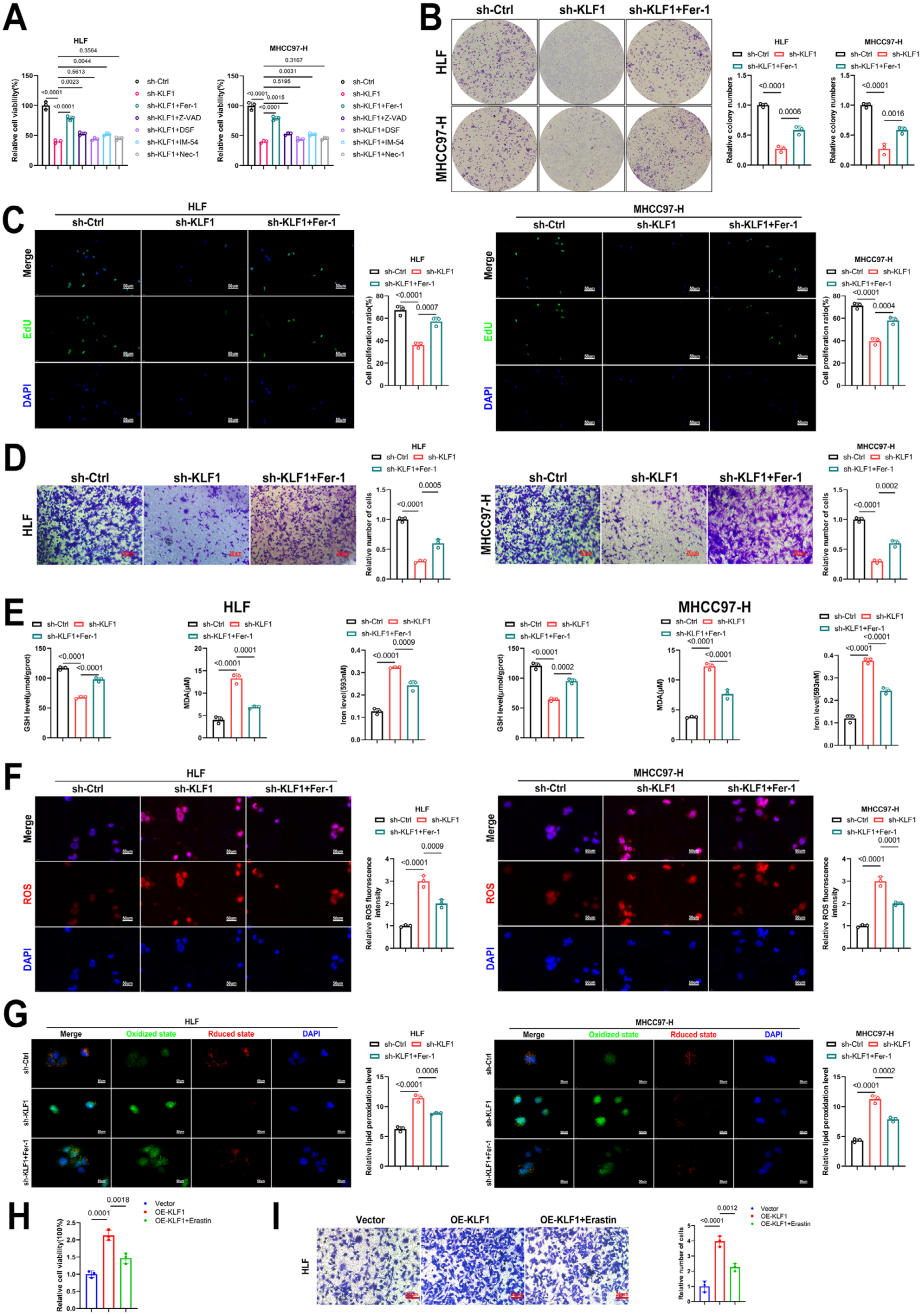
Ferroptosis modulation within HCC cells is able to restore KLF1 function. (A) The impact of various cell death inhibitors was evaluated with the CCK‐8 assay (*n* = 3). (B, C) Colony formation and EdU assays underwent performance to assess the proliferative ability of the cells (*n* = 3). (D) Movement potential within HCC tumours underwent assessment through Transwell assays (*n* = 3). (E) GSH, MDA, as well as iron levels within HCC cells were measured (*n* = 3). (F) Sample visualisations showing ROS content within HCC cells along with their corresponding quantitative analysis (*n* = 3). (G) C11 BODIPY staining images from cells are shown (*n* = 3). (H) The impact of various cell death inhibitors was evaluated with the CCK‐8 assay (*n* = 3). (I) Movement potential within HLF cells underwent assessment through Transwell assays (*n* = 3). Data are shown as mean ± SEM, and *p*‐values underwent calculation through two‐way ANOVA followed by Tukey's multiple comparisons test.

To further confirm that KLF1 influences HCC cells by regulating ferroptosis, we performed a ferroptosis inhibition experiment. Initially, after knocking down KLF1, we treated the cells with a common inhibitor of cell death and conducted the CCK8 assay. The data revealed that Ferrostatin‐1, a ferroptosis inhibitor, significantly reversed the reduction in cell viability induced by KLF1 knockdown (Figure 5A). Additionally, data from colony formation, EdU, as well as Transwell assays showed that Ferrostatin‐1 treatment enhanced growth and movement abilities in HCC cells following KLF1 knockdown (Figure 5B–D). Furthermore, Ferrostatin‐1 treatment lowered iron as well as MDA levels within HCC cells after KLF1 knockdown, whereas GSH levels were increased (Figure 5E). The ROS staining data demonstrated that Ferrostatin‐1 significantly reduced the elevated ROS levels caused by KLF1 knockdown in HCC cells (Figure 5F). C11 BODIPY staining further revealed that Ferrostatin‐1 treatment diminished lipid peroxidation levels in HCC cells after KLF1 knockdown (Figure 5G). Additionally, we further validated our findings using the ferroptosis inducer Erastin and discovered that Erastin treatment can attenuate the promoting effects of KLF1 overexpression on both the proliferation and migration capabilities of HLF cells (Figure 5H,I). In conclusion, modulating ferroptosis within HCC cells is capable of rescuing KLF1 function.

### 3.6 KLF1 Suppresses Ferroptosis by Modulating ACSL4 Transcription

**FIGURE 6:**
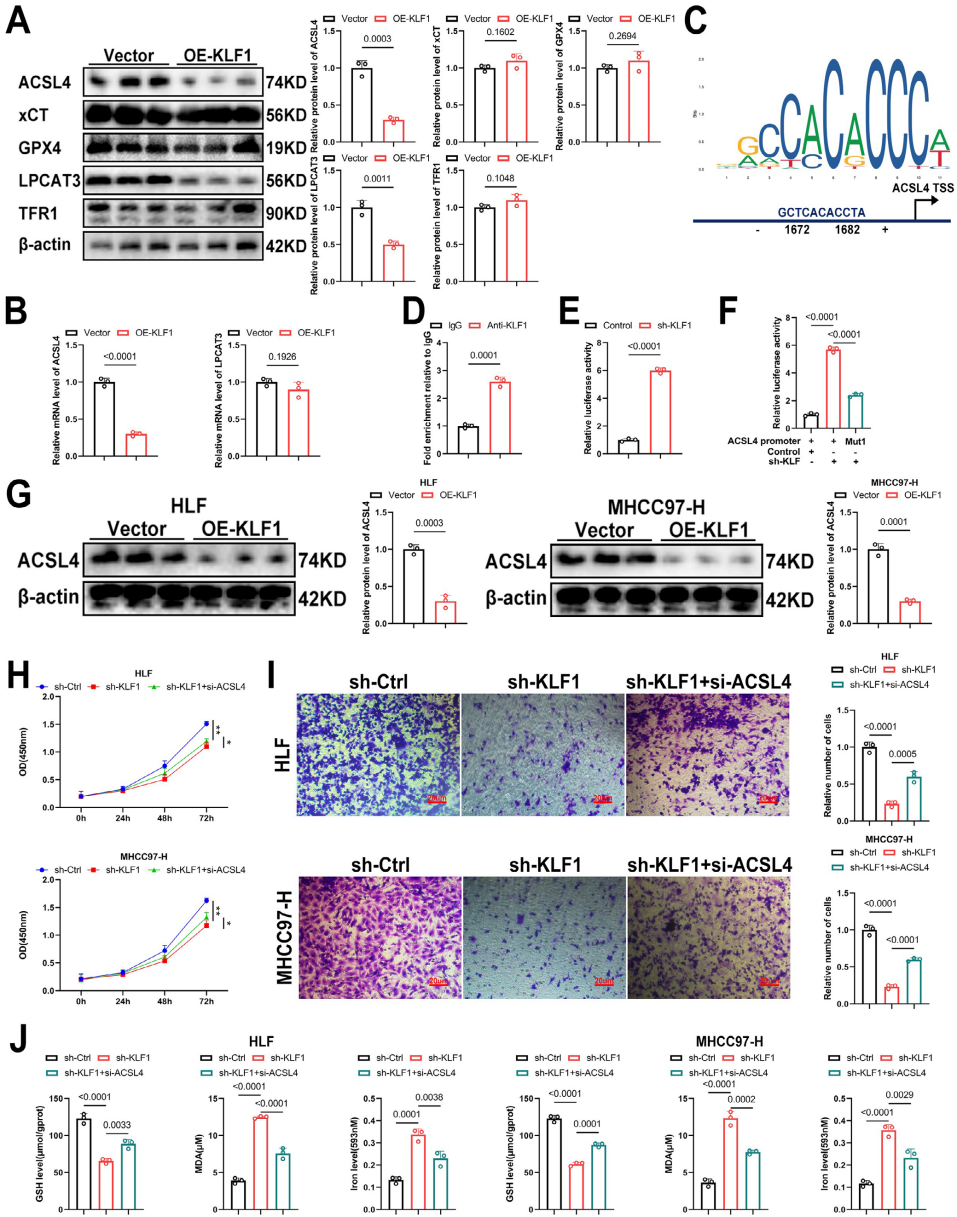
KLF1 regulates ferroptosis through the modulation of ACSL4 transcription. (A) Western blotting served for evaluating protein levels associated with ferroptosis‐related markers GPX4, xCT, ACSL4, LPCAT3, and TFR1 (*n* = 3). (B) RT‐qPCR underwent quantification for mRNA levels associated with ACSL4 as well as LPCAT3 (*n* = 3). (C, D) CHIP‐qPCR underwent use for assessing whether KLF1 binds to the ACSL4 promoter region. (E) After transfecting the cells with either the pGL3‐luciferase empty vector or the pGL3‐acsl4 luciferase construct, luciferase assays were carried out in both control and KLF1 knockdown cells. (F) Site‐directed mutagenesis was performed on the KLF1 binding site in the ACSL4 promoter. Luciferase assays were then used to assess promoter activity in cells with or without KLF1 knockdown. ACSL4 promoter activity was suppressed following mutation at site 1 (Mut1). (G) Western blotting confirmed the knockdown efficiency of ACSL4 (*n* = 3). (H) The CCK‐8 assay underwent evaluation for cellular proliferation after ACSL4 knockdown. (I) Transwell assays underwent performance for evaluating the migration capacity in cells following ACSL4 knockdown. (J) Levels of GSH, MDA, and iron in HCC cells were quantified (*n* = 3). Data are expressed as mean ± SEM, and *p*‐values were determined using two‐way ANOVA followed by Tukey's multiple comparisons test.

To investigate the mechanism by which KLF1 regulates ferroptosis, we employed Western blotting to examine key proteins involved in three crucial ferroptosis pathways: GPX4 and xCT (antioxidant system), ACSL4 and LPCAT3 (lipid peroxidation system), and TFR1 (iron transport). The results demonstrated that only ACSL4 and LPCAT3, which are vital components of the lipid peroxidation pathway, were significantly reduced after KLF1 overexpression (Figure 6A). However, RT‐qPCR analysis showed a significant reduction regarding ACSL4 mRNA expression specifically following KLF1 overexpression (Figure 6B). Since KLF1 functions as a transcription factor, we proposed that KLF1 regulates ferroptosis by modulating the transcription of ACSL4. To validate this hypothesis, CHIP‐qPCR experiments were conducted, confirming that KLF1 binds directly to the ACSL4 promoter region (Figure 6C,D). In parallel, we developed a plasmid which harbours the ACSL4 regulatory region linked to luciferase. Luciferase assays verified KLF1's inhibition on ACSL4 transcription (Figure 6E), and mutation at site 1 effectively prevented KLF1 from repressing ACSL4 transcription (Figure 6F). Subsequently, a Functional complementation experiment was carried out by knocking down ACSL4, with knockdown effectiveness verified through Western blot (Figure 6G). The CCK8 assay revealed that knocking down ACSL4 promoted cellular growth in comparison with sh‐KLF1 group (Figure 6H). Furthermore, Transwell assays showed that ACSL4 knockdown enhanced cell migration (Figure 6I). Finally, ACSL4 knockdown reduced the production of iron and MDA, while decreasing GSH levels (Figure 6J). These results suggest that KLF1 suppresses ferroptosis by inhibiting ACSL4 transcription.

### 3.7 In Vivo, KLF1 Knockdown Suppresses HCC Growth Through the ACSL4/LPCAT3 Pathway

**FIGURE 7:**
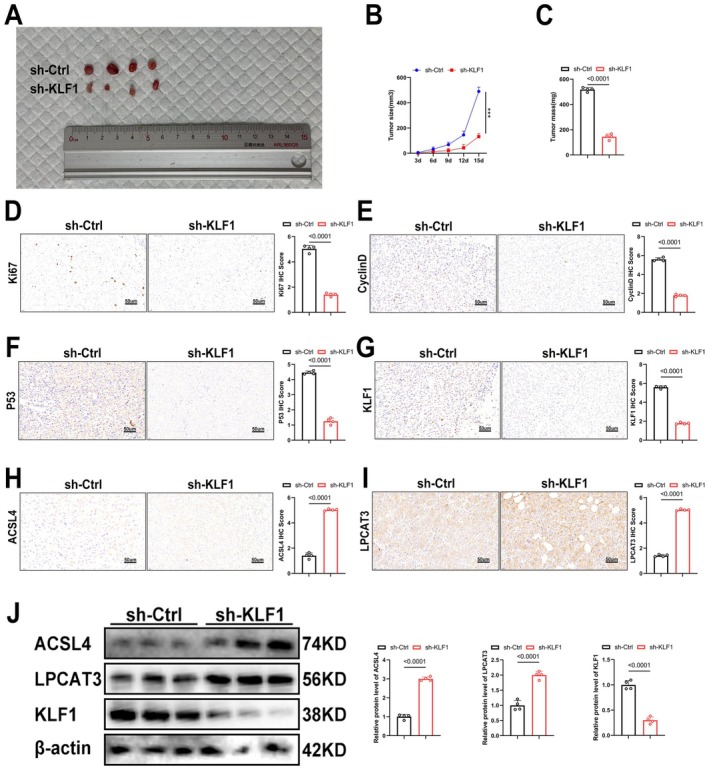
In vivo, KLF1 knockdown inhibits HCC growth through the ACSL4/LPCAT3 axis. (A, B) Images of tumours and statistical analysis of their weight (*n* = 4). (C) Tumour growth curve. (D–I) Immunohistochemical evaluation of the expression levels of Ki67, P53, CyclinD, KLF1, ACSL4, and LPCAT3 in tumour tissues from both groups (*n* = 4). (J) Western blot analysis underwent evaluation for assessing expression levels associated with KLF1, LPCAT3, as well as ACSL4 within tumour samples (*n* = 4). Data are displayed as mean ± SEM. *p*‐values underwent determination through two‐tailed independent *t*‐tests.

To further confirm our findings, we performed in vivo experiments, where we observed a significant reduction in tumour growth following KLF1 knockdown (Figure 7A–C). Analysis of tumour tissues by immunohistochemistry and Western blot revealed that KLF1 knockdown resulted in reduced levels within cell growth markers (Ki67, P53, CyclinD) as well as KLF1, while expression levels of ACSL4 and LPCAT3 were elevated (Figure 7D–J). These results suggest that KLF1 knockdown suppresses HCC growth via modulation of the ACSL4/LPCAT3 pathway in vivo.

## 4 Discussion

KLF1, a member of the KLF family, plays a pivotal role in the regulation of tumour progression. Previous studies have shown that KLF1 is overexpressed in various types of cancer, such as bladder cancer [17], leukaemia [18], and gastric cancer [19]. In addition, increased KLF1 expression in tumours is often linked to a poorer prognosis. In our research, we demonstrate that KLF1 levels are upregulated within both HCC cultures as well as samples. Elevated KLF1 levels are associated with an unfavourable outcome for HCC individuals, and silencing KLF1 significantly hampers HCC progression in both in vivo and in vitro models.

Ferroptosis, a recently discovered form of cell death, has been thoroughly investigated in hepatocellular carcinoma (HCC) and is considered a key factor in both onset as well as advancement in this condition [20, 21, 22]. The results from this study indicate KLF1 is essential for preventing ferroptosis in HCC cells by lowering intracellular iron content, reducing lipid peroxidation, and decreasing oxidative stress, while also boosting glutathione levels. The overexpression of ACSL4 and LPCAT3 has been identified as a central mechanism in ferroptosis, promoting lipid peroxidation and further driving ferroptosis. Our findings suggest that KLF1 modulates ferroptosis by suppressing ACSL4 transcription in HCC cells.

The results indicate KLF1 suppresses ferroptosis within HCC through suppression of the ACSL4/LPCAT3 signalling pathway, which consequently promotes tumour progression.

This study represents the first demonstration that KLF1 suppresses ACSL4 expression in HCC via transcriptional inhibition, thereby inhibiting ferroptosis and promoting tumour progression. However, several limitations remain in our work. For example, although we have identified KLF1 as a crucial target and established its potential value for future targeted therapies, we have not yet confirmed whether drugs specifically targeting KLF1 can effectively inhibit HCC progression. Furthermore, while we have shown that KLF1 promotes HCC by inhibiting ferroptosis, we have yet to investigate its other regulatory mechanisms, including its potential influence on glycolysis and other metabolic pathways. Finally, we did not further investigate whether KLF1 transcriptionally regulates ACSL4, which will be the focus of our future research.

Future research should focus on designing drugs that target KLF1 and conducting clinical validation. Additionally, further investigation is needed to explore other potential mechanisms by which KLF1 regulates liver cancer.

In summary, our study demonstrates the role of KLF1 in HCC progression and sheds light on the underlying mechanism, underscoring the potential of targeting KLF1 as a therapeutic strategy for future HCC treatment.

## Author Contributions

Zhihui Chen, Changyan Zhang, Jialin Yang, Yong Peng wrote the paper. Zhihui Chen offered the funding.

## Funding

The authors have nothing to report.

## Ethics Statement

This study adhered to the principles outlined in the Helsinki Declaration and received approval from the Medical Ethics Committee of Nanchong Central Hospital.

## Consent

The authors have nothing to report.

## Conflicts of Interest

The authors declare no conflicts of interest.

## Data Availability

The data that support the findings of this study are available from the corresponding author upon reasonable request.
